# Community structures of mangrove endophytic and rhizosphere bacteria in Zhangjiangkou National Mangrove Nature Reserve

**DOI:** 10.1038/s41598-023-44447-2

**Published:** 2023-10-10

**Authors:** Zongsheng Yuan, Zhihao Zeng, Fang Liu

**Affiliations:** 1 College of Geography and Oceanography, Minjiang University, Fuzhou, Fujian China; 2 College of Life Sciences, Fujian Agriculture and Forestry University, Fuzhou, Fujian China

**Keywords:** Microbiology, Environmental sciences

## Abstract

Bacterial communities play an important role in mangrove ecosystems. In order to gain information on the bacterial communities in mangrove species and rhizospheres grown in Zhangjiangkou National Mangrove Nature Reserve, this study collected root, branch, and leaf samples from five mangrove species as well as rhizosphere and non-rhizosphere samples and analyzed the community structure of endophytic bacteria and bacteria in rhizosphere and non-rhizosphere using Illumina high-throughput sequencing technique. Bacteria in 52 phyla, 64 classes, 152 orders, 295 families, and 794 genera were identified, which mainly belonged to Proteobacteria, Cyanobacteria, Actinobacteria, Firmicutes, Bacteroidetes, Fusobacteria, and Nitrospirota. At each taxonomic level, the community structure of the rhizosphere bacteria varied slightly with mangrove species, but endophytic bacteria differed greatly with plant species. The diversity indices of endophytic bacteria in branch and leaf samples of *Acanthus ilicifolius* were significantly lower, and endophytic bacteria in the plant tissues had higher abundance in the replication/repair and translation Clusters of Orthologous Genes functional categories but lower abundance in the carbohydrate metabolism category. This study helps to understand the community structure and diversity characteristics of endophytic and rhizosphere bacteria in different mangrove plants. Provide a theoretical basis for in-depth research on the functions of mangrove ecosystems.

## Introduction

Mangrove forests are unique wetland ecosystems distributed along tropical and subtropical coastlines and are of considerable importance in maintaining sea levels and protecting coasts^1^. Mangrove ecosystems are considered important coastal carbon pools and provide abundant nutrients for microorganisms, plants, and wetland animals^2^. Microorganisms are a crucial component of mangrove ecosystems and play an important role in promoting carbon, nitrogen, and phosphorus cycling^3^. The diversity and composition of microbial communities in mangrove ecosystems are largely influenced by mangrove plant species^4^ and environments^5^, and microbial community composition may vary with mangrove plant species^6^. At present, most of the research on the microbial community structure of mangrove plants has focused on the rhizosphere bacteria, and the composition of mangrove endophytic bacteria has rarely been reported. In addition, there is a lack of comprehensive comparisons of the microbial community compositions associated with different mangrove species.

Zhangjiangkou National Mangrove Nature Reserve, Ramsar site no. 1726, is the largest *Avicennia marina* forest in China^7^, located in the Zhangzhou city, Fujian province. The mangrove forests at this reserve mainly include *Kandelia candel*, *K. obovata*, and *Bruguiera gymnorrhiza*, which belong to the family Rhizophoraceae; *Avicennia marina* and *Acanthus ilicifolius*, two species of the family Acanthaceae; and *Aegiceras corniculatum*, a member of the family Primulaceae^8^. Thus far, there is little information on endophytic bacteria and the microbial community compositions of these species. In the present study, we hypothesized that community compositions of both endophytic and rhizosphere bacteria could vary with plant species, and the identification of endophytic and rhizosphere bacteria and microbial composition would provide much need information for further investigation of their role in maintaining mangrove ecosystem.

Traditional investigation of microbial community relies on sampling, laboratory cultivation, and microscopic identification if microbes can be cultivated^9^. Currently, high-throughput sequencing has been widely used for the in-depth study of microbial community composition, which provides taxonomic resolution down to the level of operational taxonomic units (OTS) and permit analysis of changes in community composition^4,10,11^. Most of the microbial communities in mangrove plants consist of bacteria^12–14^, which play an important role in nutrient transformation in mangrove ecosystems^3^.

The objectives of this study were to analyze the composition and diversity of endophytic bacteria in five representative mangrove species as well as their rhizospheres bacterial communities by means of Illumina high throughput sequencing^15^ and to test if endophytic and rhizosphere bacterial community composition differences were associated with plant species in this Reserve.

## Results

### Sequencing results

**Figure 1:**
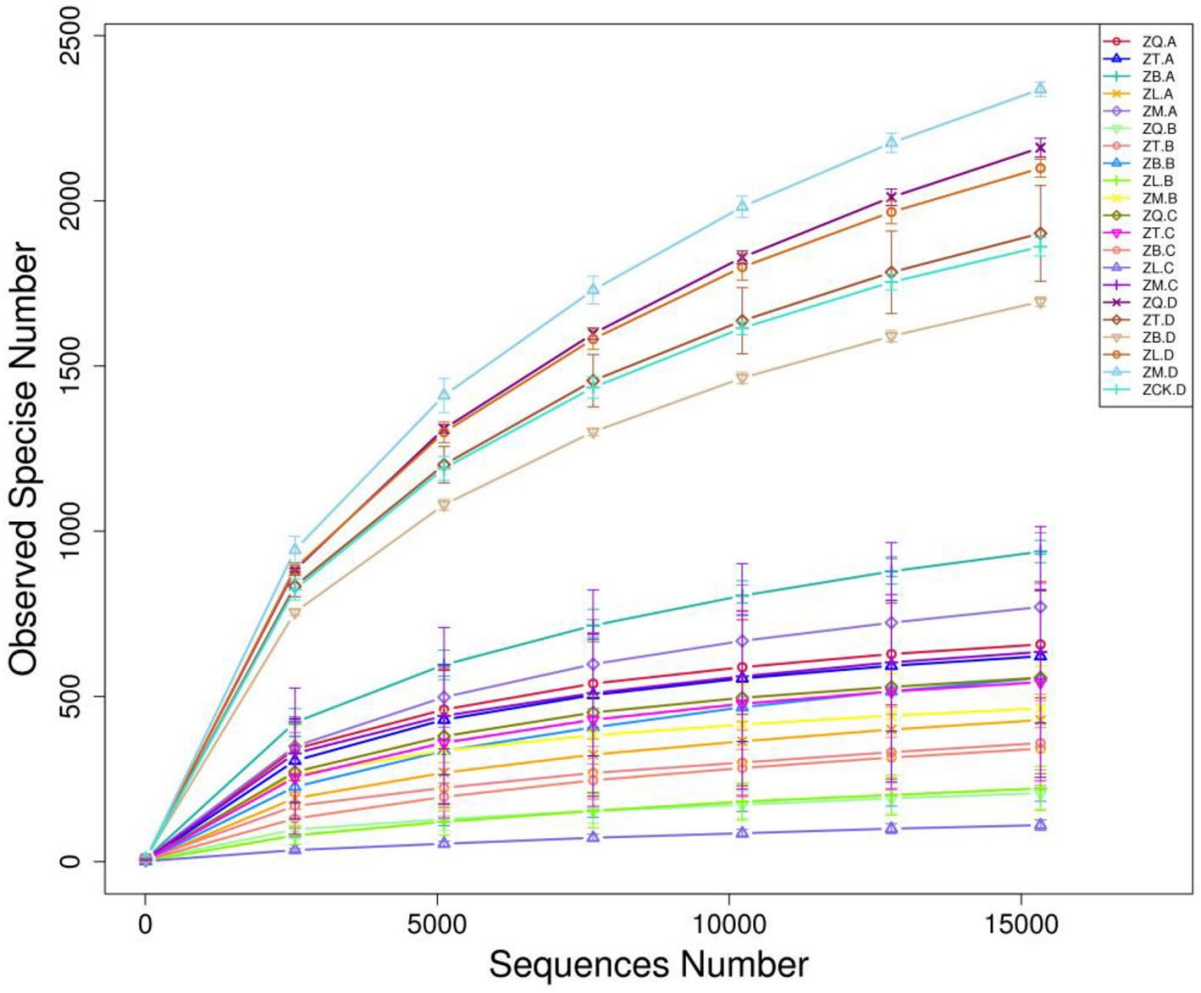
Rarefaction curves for plant samples of five mangrove species and their rhizospheres and non-rhizosphere at the 97% similarity level, where ZQ, ZT, ZB, ZL, and ZM represent *K. candel*, *A. corniculatum*, *A. marina*, *A. ilicifolius*, and *B. gymnorrhiza*, respectively A, B, C, and D after the species symbol indicate root, branch, leaf, and soil samples, and ZCK.D is non-rhizosphere sample.

The V5–V7 regions of bacterial 16S rRNA gene were amplified using the DNA prepared from the roots, branches, and leaves of the trees of five mangrove species as well as rhizosphere and non-rhizosphere samples. Following high-throughput sequencing, a total of 4,956,926 valid sequences were obtained, of which the average length was 383 bp, and the library coverage was 0.942–0.996. The data shown in Fig. 1 revealed that as the number of sequences increased, the rarefaction curves for different samples gradually reached a plateau, indicating that in this study the number of sequences was close to saturation and the sequencing data were reasonable and capable of reflecting the structure of most of the bacterial communities in the samples.

### Composition of bacterial communities

**Figure 2:**
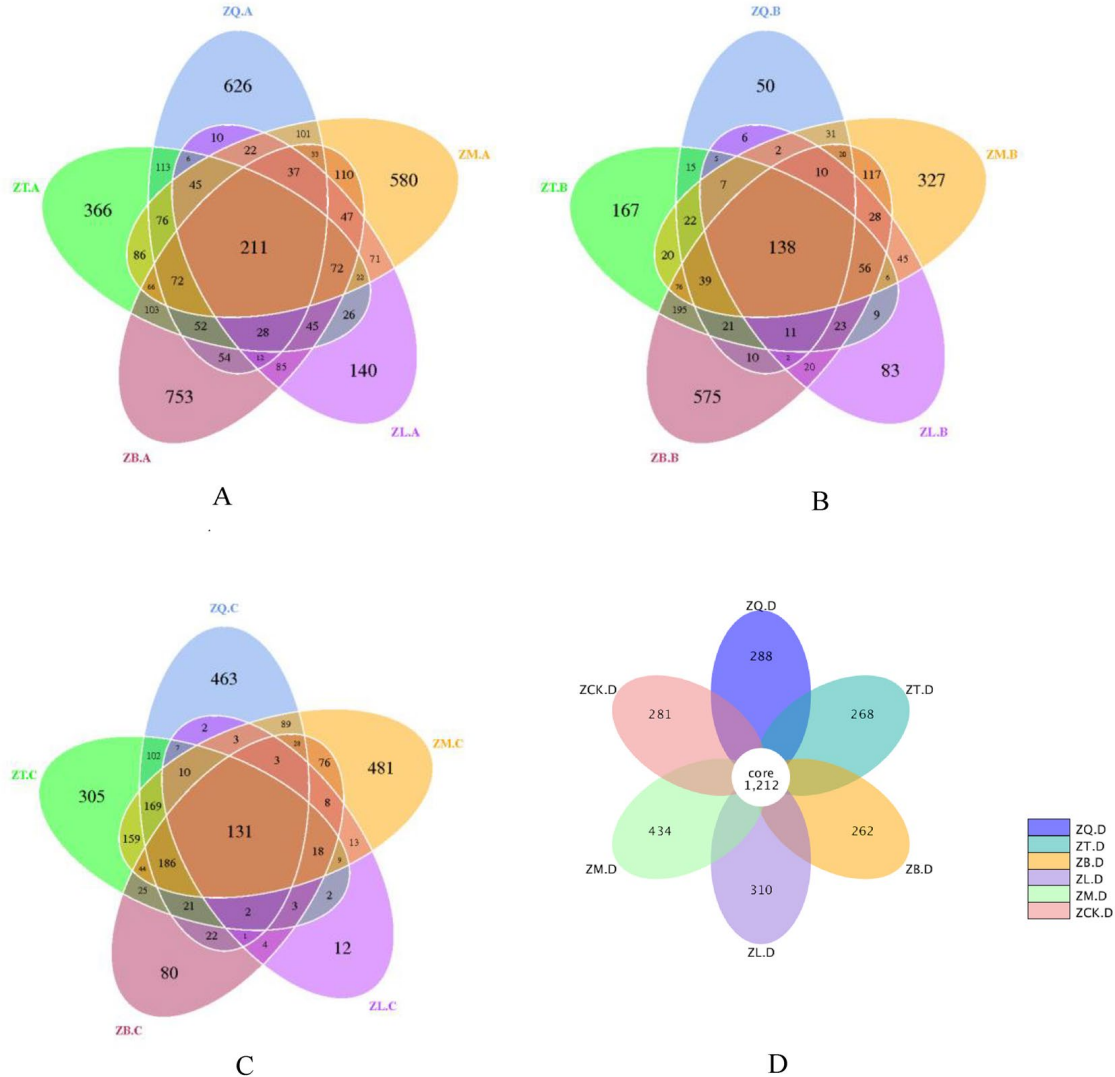
Distribution of the number of operational taxonomic units (OTUs) among the plant tissue samples of five mangrove species and soil samples (**A**: root sample; **B**: branch sample; **C**: leaf sample; and **D**: soil sample), where ZQ, ZT, ZB, ZL, and ZM represent *K. candel*, *A. corniculatum*, *A. marina*, *A. ilicifolius*, and *B. gymnorrhiza*, respectively.

A total of 52 phyla, 64 classes, 152 orders, 295 families, and 794 genera of bacteria were identified in 63 samples collected from five mangrove species and related soils. Figure 2 shows the number of common OTUs shared among different samples. The roots (Fig. 2A), branches (Fig. 2B), leaves (Fig. 2C), and rhizospheres (Fig. 2D), related to five mangrove species shared 211, 138, 131, and 1212 common OTUs, respectively. Among them, soil samples had the largest number of shared OTUs, followed by root samples. Branch and leaf samples had a similar number of common OTUs. The different numbers of common OTUs from samples reflected the differences among them, specifically there were small differences between the soil samples but distinct differences between the branch and leaf samples for all five mangrove species.

**Figure 3:**
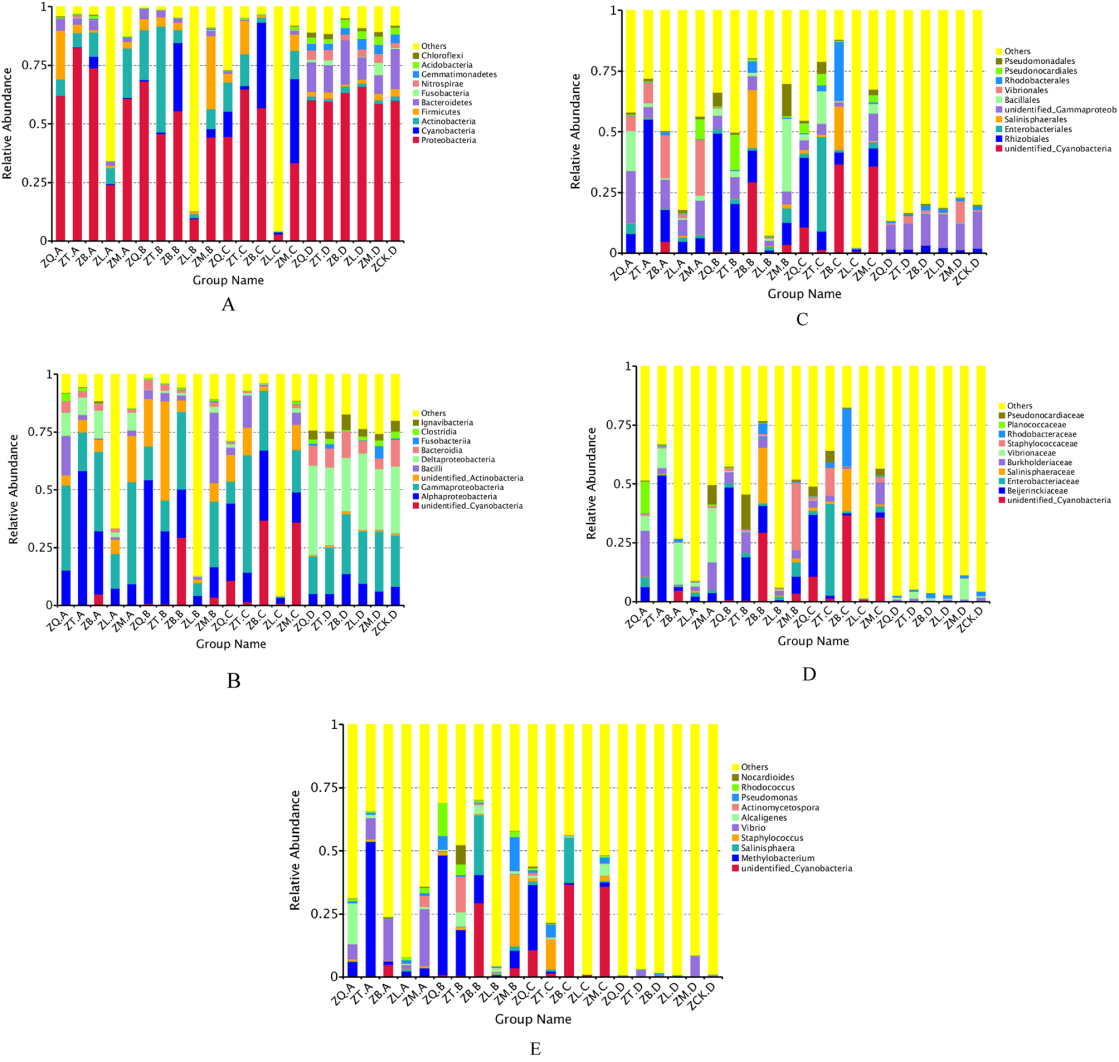
Relative abundances of the bacterial populations in the plant tissue samples of five mangrove species and soil samples. For the group name, ZQ, ZT, ZB, ZL, and ZM represent *K. candel*, *A. corniculatum*, *A. marina*, *A. ilicifolius*, and *B. gymnorrhiza*, respectively and A, B, C, and D after the species symbol indicate root, branch, leaf, and soil samples, and ZCK.D is non-rhizosphere sample (**A**: phylum level; **B**: class level; **C**: order level; **D**: family level; **E**: genus level).

At the phylum level (Fig. 3A), the bacteria in root, branch, leaf, and rhizosphere of the five mangrove species as well as non-rhizosphere mainly included the Proteobacteria, Cyanobacteria, Actinobacteria, Firmicutes, Bacteroidetes, Fusobacterium, Nitrospirae, Germmatimonadetes, Acidobacteria, and Chloroflexi phyla, of which the Proteobacteria was the dominant phylum. The second most dominant bacterial phylum in root sample ZQ.A of *K. candel* was Firmicutes (20.82%), and the second most dominant phylum in the roots of the other four species of mangrove plants was Actinobacteria. The second most dominant phylum in branch sample ZQ.B of *K. candel* and ZT.B of *A. corniculatum* was Actinobacteria, accounting for 21.29% and 45.19%, respectively. The second most dominant phylum in branch sample ZB.B of *A. marina* was Cyanobacteria (29.27%); and the second dominant phylum in branch sample ZM.B of *B. gymnorrhiza* was Firmicutes (31.28%). The dominant phyla in leaf sample ZQ.C of *K. candel* and ZM.C of *B. gymnorrhiza* were Proteobacteria, Cyanobacteria, and Actinobacteria; the dominant phyla in leaf sample ZT.C of *A. corniculatum* were Proteobacteria, Actinobacteria, and Firmicutes; and the dominant phyla in leaf sample ZB.C were Proteobacteria and Cyanobacteria. For the soil samples, the abundance of bacteria in Bacteroidetes in the rhizosphere sample ZB.D from *A. marina* was higher than that in the control ZCK.D (non-rhizosphere); however, the overall difference in the bacterial compositions among the soil samples regardless of rhizosphere or non-rhizosphere were rather small.

With regard to the bacteria at the class level (Fig. 3B), bacterial communities in the root, branch, and leaf samples of the five mangrove species and their rhizospheres were dominated by Alphaproteobacteria and Gammaproteobacteria. Root sample ZQ.A of *K. candel* included a proportion of Bacilli (17.27%). Gammaproteobacteria accounted for 12.03% of the bacteria in root sample ZB.A of *A. marina*. A proportion of unidentified bacteria in the Actinobacteria phylum was found in the branch sample of ZQ.B and leaf sample of ZQ.C (*K. candel*) as well as in branch sample ZT.B and leaf sample ZT.C of *A. corniculatum*. The unidentified bacteria in the Cyanobacteria phylum accounted for 29.27% and 36.68% of the bacteria in branch sample ZB.B and leaf sample ZB.C of *A. marina*, respectively. The proportions of the bacteria in the Bacilli class and the unidentified bacteria in the Cyanobacteria phylum were 30.51% and 35.95% in branch sample ZM.B and leaf sample ZM.C of *B. gymnorrhiza*, respectively. The bacterial communities in the soil samples collected from the mangrove forests were dominated by bacteria in the Gammaproteobacteria class and Deltaproteobacteria class. At the class level, there was minor difference in soil bacterial community compositions irrespective of where the soils were collected.

At the order level (Fig. 3C), the bacterial communities in root sample ZT.A of *A. corniculatum* and root sample ZB.A of *A. marina* were dominated by bacteria in the Rhizobiales and Vibrionales. The most abundant bacterial order in root sample ZQ.A of *K. candel* were Rhizobiales, unidentified bacteria in the Gammaproteobacteria class, and Bacillales. The prevailing bacterial orders in root sample ZM.A of *B. gymnorrhiza* were Rhizobiales, unidentified bacteria in the Gammaproteobacteria class, and Vibrionales. The dominant bacterial orders in root sample ZL.A of *A. ilicifolius* were Rhizobiales and unidentified bacteria in the Gammaproteobacteria class. Rhizobiales were found to be the most abundant bacterial order in the branches of mangrove plants but varied with mangrove species. The proportion of Pseudonocardiales was 14.46% in the bacterial community in branch sample ZT.B of *A. corniculatum*. The percentage of Salinisphaerales in the bacterial community in branch sample ZB.B of *A. marina* was 23.73%. Bacillales and Pseudomonadales accounted for 29.86% and 13.30% of the bacterial community in branch sample ZM.B of *B. gymnorrhiza*, respectively. Rhizobiales and unidentified bacteria in the Cyanobacteria phylum dominated the bacterial community in the leaf sample ZQ.C of *K. candel*. Enterobacterales and Bacillales dominated the bacterial community in leaf sample ZT.C of *A. corniculatum*. Rhodospirillales and Salinisphaerales were the dominant bacterial orders in leaf sample ZB.C of *A. marina.* Unidentified bacteria in the Cyanobacteria phylum and the Gammaproteobacteria class dominated the bacterial community in leaf sample ZM.C of *B. gymnorrhiza*. At the order level, the differences in bacterial community composition in the soil samples from different mangrove species again were small, and the majority of the bacteria were in the unidentified Gammaproteobacteria class and Rhizobiales. Vibrionales accounted for 8.86% of the bacteria in the soil samples related to *B. gymnorrhiza*, which was the highest proportion compared with other four mangrove species.

For bacteria at the family level (Fig. 3D), the dominant bacterial families were Burkholderiaceae (19.54%) and Planococcaceae (13.49%) in root sample ZQ.A of *K. candel*. The prevailing bacterial families in root sample ZT.A of *A. corniculatum* were Beijerinckiaceae (53.45%) and Pseudonocardiaceae (14.46%). The abundant bacterial family in root sample ZB.A of *A. marina* was Vibrionaceae (17.67%). The dominant bacterial families in root sample ZM.A of *B. gymnorrhiza* were Burkholderiaceae (11.70%) and Vibrionaceae (23.10%). Beijerinckiaceae was the most abundant in branch sample ZQ.B of *K. candel*, accounting for 47.75% of bacterial families. The dominant bacterial family in branch sample ZT.B of *A. corniculatum* was Beijerinckiaceae (18.29%). The prevailing bacterial families in branch sample ZT.B of *A. marina* were Beijerinckiaceae (11.42%) and Salinisphaeraceae (23.73%). The dominant bacterial family in branch sample ZM.B of *B. gymnorrhiza* was Staphylococcaceae (28.58%). Beijerinckiaceae (26.27%) was the most abundant bacterial family in leaf sample ZQ.C of *K. candel*. The dominant bacterial families in leaf sample ZT.C of *A. corniculatum* were Enterobacteriaceae (38.86%) and Staphylococcaceae (11.86%). The prevailing bacterial families in leaf sample ZB.C of *A. marina* were Salinisphaeraceae (18.06%) and Rhodospirillaceae (24.48%). The dominant bacterial family in leaf sample ZM.C of *B. gymnorrhiza* was Burkholderiaceae (9.56%). For all the soil samples regardless of rhizosphere or non-rhizosphere, the identified bacteria at the family level had low abundance and low difference in community compositions.

At the genus level (Fig. 3E), the dominant bacterial genera and proportions in the tissue samples of mangrove plants were as follows: 16.24% of *Alcaligenes* in root sample ZQ.A of *K. candel*; 53.44% of *Methylobacterium* in root sample ZT.A of *A. corniculatum*; 16.51% of *Vibrio* in root sample ZB.A of *A. marina*; 22.61% of *Vibrio* in root sample ZM.A of *B. gymnorrhiza*. Additionally, *Methylobacterium* (47.49%) and *Rhodospirillum* (12.93%) in branch sample ZQ.B of *K. candel*; *Methylobacterium* (18.14%) and *Actinomycetospora* (14.23%) in branch sample ZT.B of *A. corniculatum*; *Methylobacterium* (11.18%) and *Salinisphaera* (23.73%) in branch sample ZB.B of *A. marina*; and *Staphylococcus* (28.58%) and *Pseudomonas* (13.19%) in branch sample ZM.B of *B. gymnorrhiza*. For the leaf and soil samples collected from the forests of different mangrove species, the bacteria identified at the genus level had low abundance and there were no distinct differences in bacterial abundance among the samples.

### Alpha diversity of bacterial communities

The alpha diversity indices calculated based on the number of OTUs of the root, branch, leaf, and soil samples collected from the five mangrove species are presented in Table 1, which were highly variable with plant species and plant organs, but less variable among rhizospheres. The inter-group differences of the alpha diversity indices among the samples are shown in Table 2. The difference between root sample ZB.A of *A. marina* and root sample ZL.A of *A. ilicifolius* was significant in the number of observed species, Chao1 index, and phylogenetic diversity (PD) index, and highly significant in the Shannon index (Table 2). There was a significant difference in Shannon index between root sample ZB.A of *A. marina* and the root sample ZT.A of *A. corniculatum*. The branch samples between ZB.B of *A. marina* and ZL.B of *A. ilicifolius* had significant differences in the number of observed species. Significant differences occurred between ZB.B of *A. marina* and ZQ,.B of *K. candel* in the number of observed species and PD index. ZL.B of *A. ilicifolius* and ZM.B of *B. gymnorrhiza* showed significantly high in the number of observed species and the phylogenetic diversity index, and highly significant in the Shannon index. The difference between branch samples of ZL.B (*A. ilicifolius*) and ZT.B (*A. corniculatum*) was highly significant in the Shannon index. Branch samples between ZQ.B of *K. candel* and ZM.B of *B. gymnorrhiza* as well as between ZQ.B of *K. candel* and ZB.B of *A. marina* had significant differences in the number of observed species and the phylogenetic diversity index. The difference between leaf sample ZL.C of *A. ilicifolius* and the leaf samples of the other four mangrove species was significant in the number of observed species, Chao1 index, Shannon index, and phylogenetic diversity index. In addition, the significant difference in soil samples occurred between ZB.D of *A. marina* and ZM.D of *B. gymnorrhiza* only in the number of observed species and PD index.

**Table 1:** Alpha diversity index of five mangrove plant and their rhizospheres samples.

Sample	Observed species	Chao1	Shannon	PD whole tree	Goods coverage
ZQ.A	657	778.47	5.602	91.881	0.99
ZT.A	621	750.281	4.134	106.729	0.99
ZB.A	938	1242.859	6.715	126.476	0.979
ZL.A	428	640.833	3.159	69.016	0.989
ZM.A	770	952.541	5.854	104.402	0.984
ZQ.B	207	342.42	3.448	36.261	0.994
ZT.B	359	564.717	4.884	57.204	0.99
ZB.B	556	803.282	3.528	79.678	0.985
ZL.B	221	388.095	1.198	39.059	0.993
ZM.B	463	614.349	4.998	74.376	0.992
ZQ.C	557	673.824	4.152	89.647	0.991
ZT.C	543	644.767	4.365	82.129	0.99
ZB.C	342	501.818	2.983	54.617	0.99
ZL.C	110	193.45	0.44	22.882	0.996
ZM.C	634	817.839	5.114	93.768	0.988
ZQ.D	2160	2936.216	9.165	216.104	0.946
ZT.D	1901	2511.561	9.009	198.5	0.958
ZB.D	1694	2114.462	8.779	181.436	0.964
ZL.D	2098	2726.854	9.115	215.193	0.952
ZM.D	2337	3150.936	9.173	231.655	0.942

For the samples, ZQ, ZT, ZB, ZL, and ZM represent *K. candel*, *A. corniculatum*, *A. marina*, *A. ilicifolius*, and *B. gymnorrhiza*, respectively and A, B, C, and D after the species symbol indicate root, branch, leaf, and soil samples, and ZCK.D is non-rhizosphere sample.

**Table 2:** Inter-group differences of Alpha diversity index of five mangrove species and their rhizospheres samples.

Inter-group	Observed species	Chao1	Shannon	Phylogenetic diversity index
ZB.A-ZL.A	*	*	**	*
ZB.A-ZT.A			*	
ZB.B-ZL.B	*			
ZB.B-ZQ.B	*			*
ZL.B-ZM.B	*		**	*
ZL.B-ZT.B			**	
ZM.B-ZQ.B	*			*
ZB.C-ZL.C	*	*		*
ZL.C-ZM.C	***	**	***	***
ZL.C-ZQ.C	**	**	**	***
ZL.C-ZT.C	**	*	**	**
ZB.D-ZM.D	*			*

ZQ, ZT, ZB, ZL, and ZM represent *K. candel*, *A. corniculatum*, *A. marina*, *A. ilicifolius*, and *B. gymnorrhiza*, respectively and A, B, C, and D after the species symbol indicate root, branch, leaf, and soil samples, and ZCK.D is non-rhizosphere sample.

**Figure 4:**
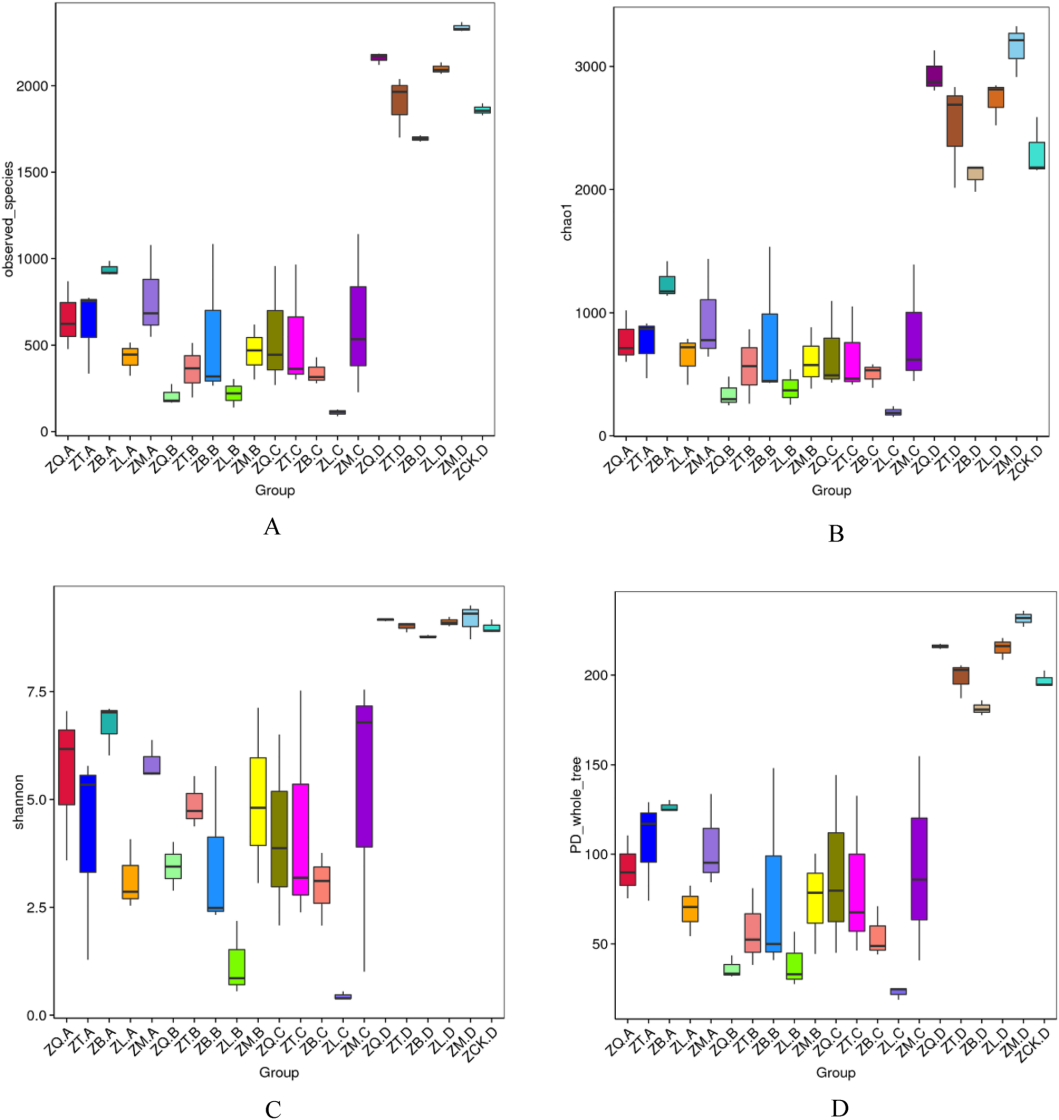
Boxplots of the diversity indices of the bacteria in the plant tissue samples of five mangrove species. (**A**) number of observed species; (**B**) Chao1 index; (**C**) Shannon index; (**D**) phylogenetic diversity index. For the label of group, ZQ, ZT, ZB, ZL, and ZM represent *K. candel*, *A. corniculatum*, *A. marina*, *A. ilicifolius*, and *B. gymnorrhiza*, respectively and A, B, C, and D after the species symbol indicate root, branch, leaf, and soil samples, and ZCK.D is non-rhizosphere sample.

Boxplots of Alpha-diversity indices are present in Fig. 4. Observed species (Fig. 4A) and Chao1 index (Fig. 4B) reflected the OUT abundance in samples. Shannon (Fig. 4C) and PD (Fig. 4D) indices represented the diversity of OUT in the samples. The greater the Chao1, the higher the expected species richness of microbiota. The greater the Shannon, the higher the diversity of microbiota.

### Beta diversity of bacterial communities

**Figure 5:**
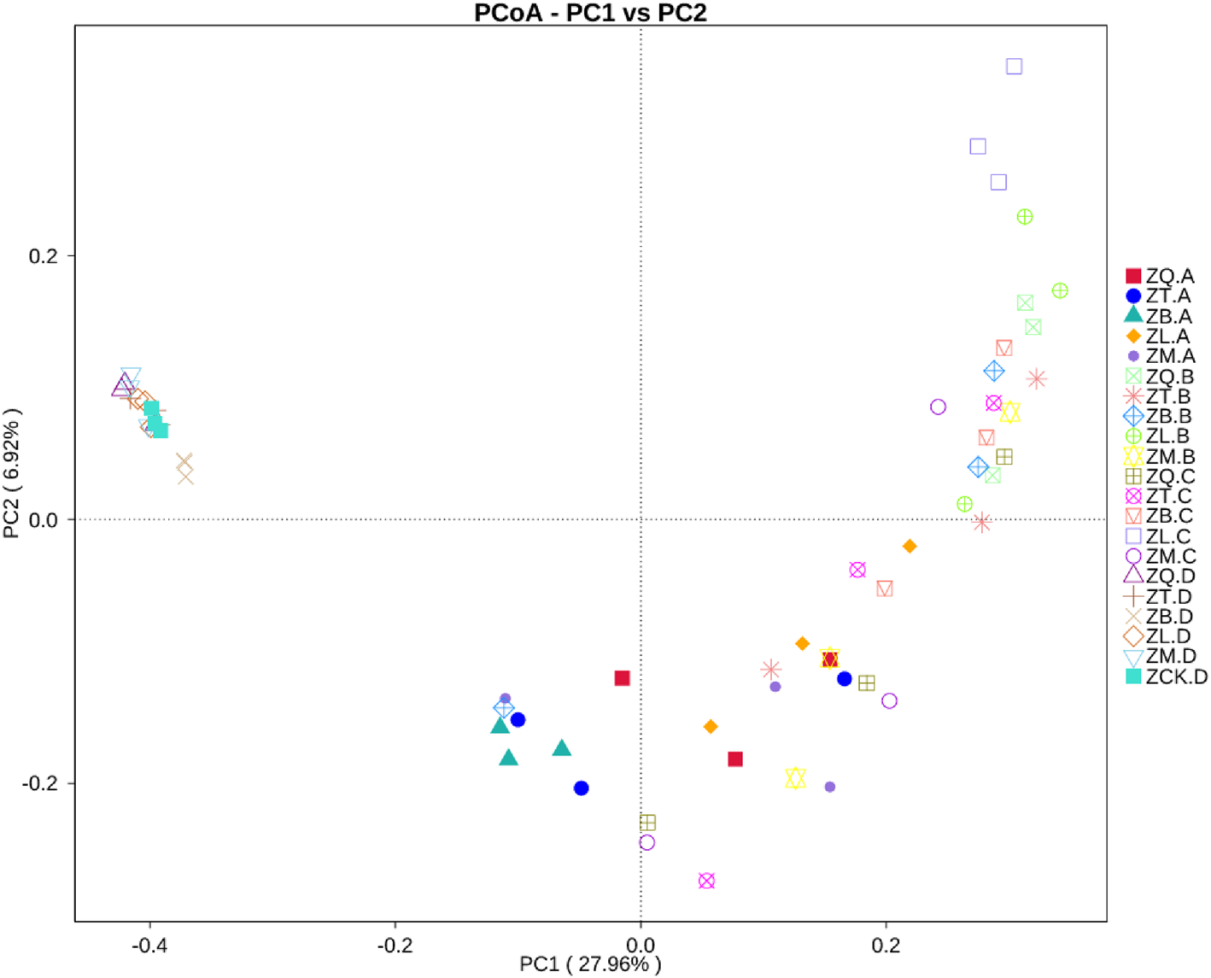
Principal coordinates analysis (PCoA) of the plant tissue samples of five mangrove species and their rhizospheres samples based on the unweighted UniFrac distance. Where ZQ, ZT, ZB, ZL, and ZM represent *K. candel*, *A. corniculatum*, *A. marina*, *A. ilicifolius*, and *B. gymnorrhiza*, respectively and A, B, C, and D after the species symbol indicate root, branch, leaf, and soil samples, and ZCK.D is non-rhizosphere sample.

**Figure 6:**
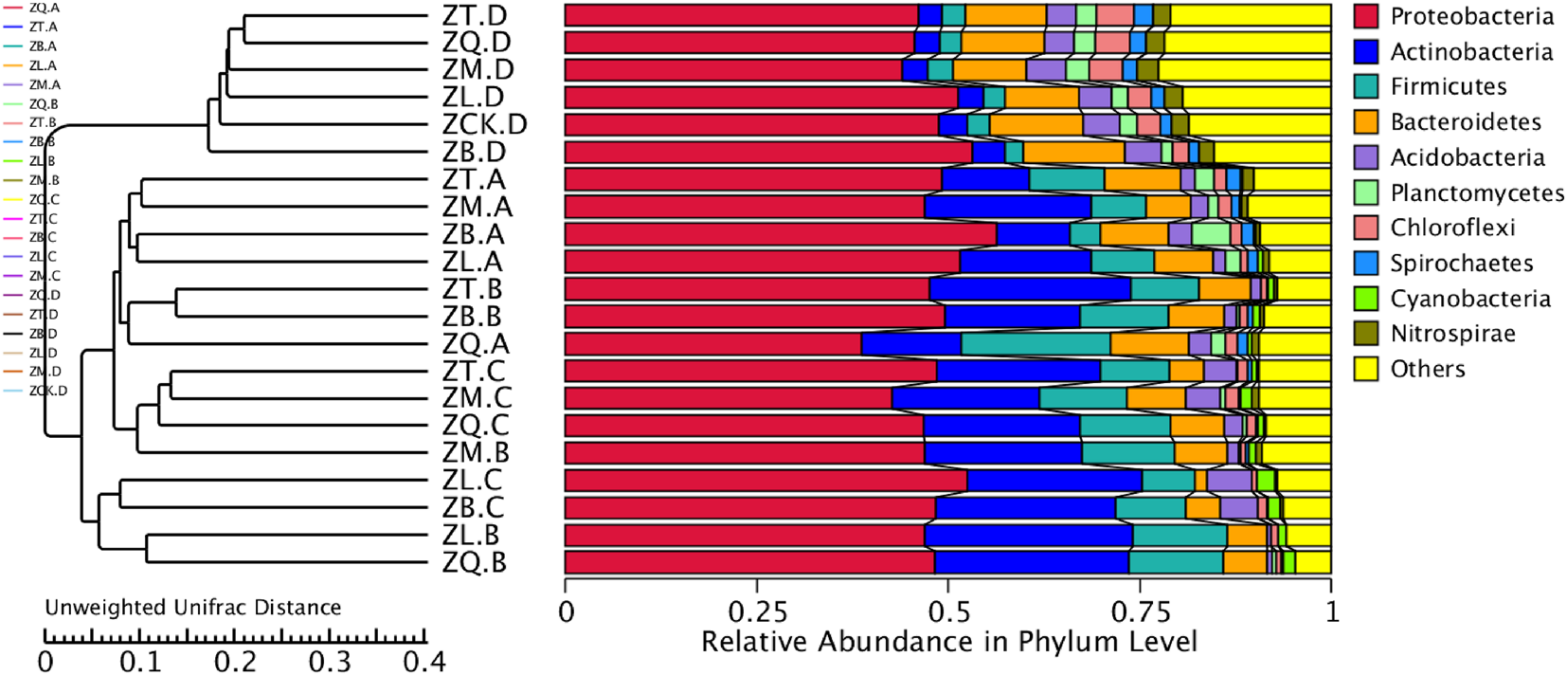
Unweighted pair group method with arithmetic mean (UPGMA) cluster tree of the plant tissue samples of five mangrove species and their rhizospheres based on the unweighted UniFrac distance.

PCoA analysis was performed based on the number of OTUs of the root, branch, leaf, and soil samples related to five mangrove species (Fig. 5). Clustering was performed to construct an unweighted pair group method with arithmetic mean (UPGMA) cluster tree of the samples (Fig. 6). The results indicated that the distance between the soil samples collected from the forests of five mangrove species was relatively small, and the UPGMA cluster tree also showed that there was no distinct difference between the soil samples, suggesting that all the soil samples had similar bacterial compositions and that the mangrove species had little effect on the soil bacterial community.

### Prediction of bacterial functions

**Figure 7:**
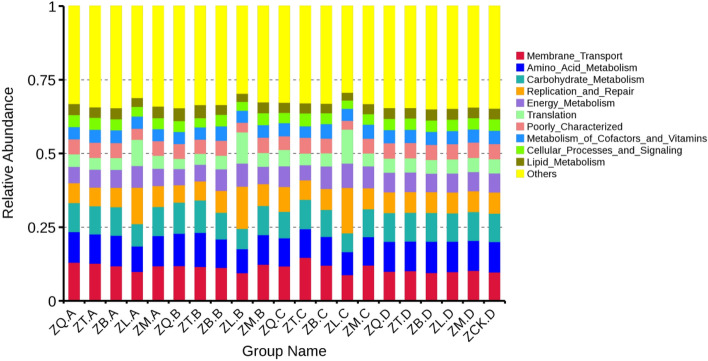
Histogram of the relative abundances of Clusters of Orthologous Genes (COG) functional categories of the dominant bacteria in the plant tissue samples of five mangrove species and their rhizospheres. Where ZQ, ZT, ZB, ZL, and ZM represent *K. candel*, *A. corniculatum*, *A. marina*, *A. ilicifolius*, and *B. gymnorrhiza*, respectively and A, B, C, and D after the species symbol indicate root, branch, leaf, and soil samples, and ZCK.D is non-rhizosphere sample.

The functions of the bacteria in the root, branch, leaf, and soil samples collected from the five mangrove species were predicted. The top 10 functional categories with the highest abundances at Clusters of Orthologous Genes (COG) level 2 were used to generate Fig. 7 showing the relative abundances of COG functional categories in the samples. The top 10 COG functional categories, namely, membrane transport, amino acid metabolism, carbohydrate metabolism, replication and repair, energy metabolism, translation, metabolism of cofactors and vitamins, cellular processes and signaling, and lipid metabolism, were roughly the same for the bacteria in all samples. There was little difference in the predicted functional categories between the samples collected from *K. candel*, *A. marina*, *A. corniculatum*, and *B. gymnorrhiza*, while the relative abundances of the replication/repair and translation categories of the bacteria in the root, branch, and leaf samples of *A. ilicifolius* were higher, and the relative abundance of the carbohydrate metabolism category in the *A. ilicifolius* tissue samples was lower compared to the other four mangrove species.

**Figure 8:**
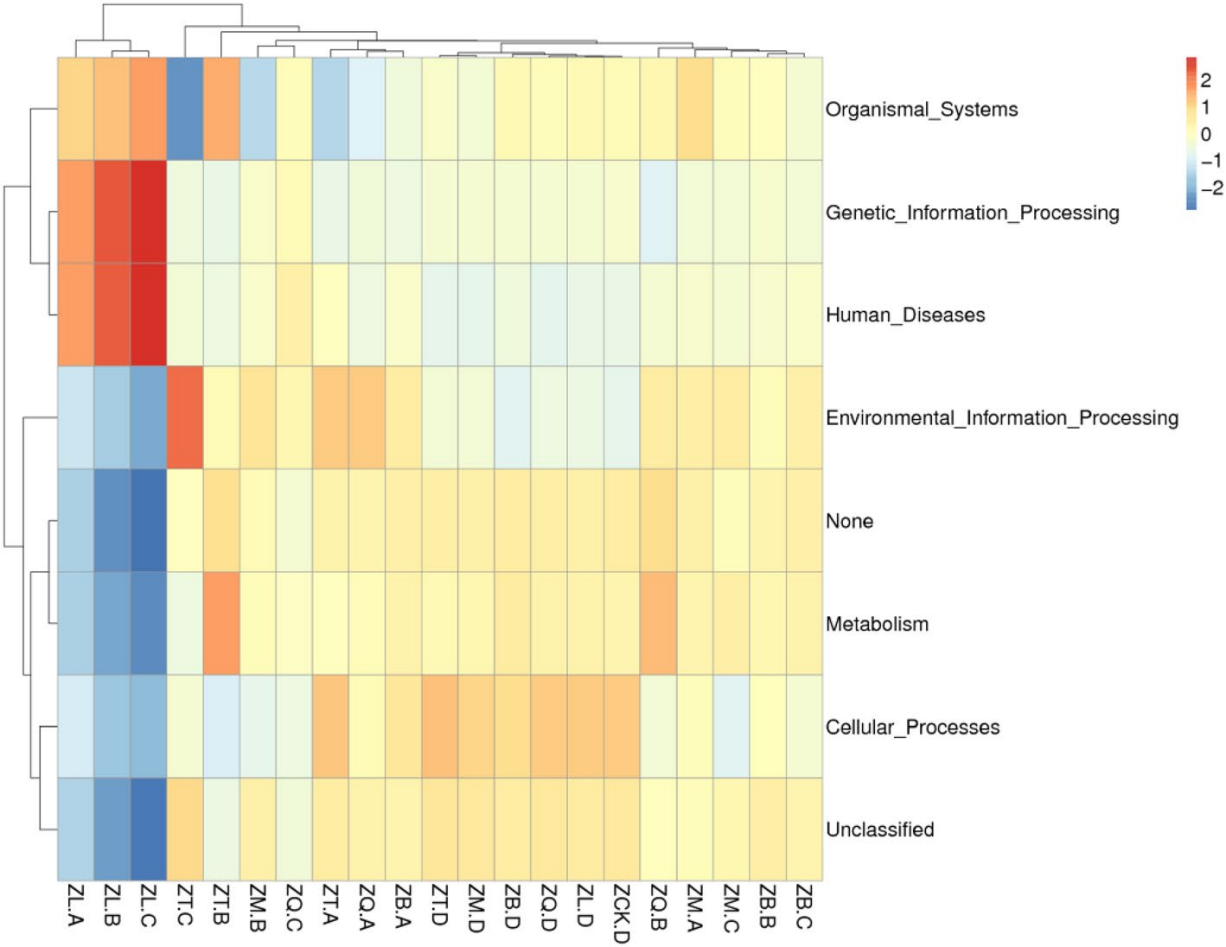
Heat map of bacterial gene functional prediction using the PICRUSt algorithm. Each row refers to a KEGG pathway, and each column is a sample where ZQ, ZT, ZB, ZL, and ZM represent *K. candel*, *A. corniculatum*, *A. marina*, *A. ilicifolius*, and *B. gymnorrhiza*, respectively and A, B, C, and D after the species symbol indicate root, branch, leaf, and soil samples, and ZCK.D is non-rhizosphere sample.

The data in Fig. 8 show that the difference between mangrove rhizosphere bacteria and endophytic bacteria was distinct in the categories of metabolism, cellular processes, and organismal systems. The endophytic bacteria in the tissue samples of *A. ilicifolius* had higher abundance in organismal systems, genetic information processing, and human diseases, while the endophytic bacteria of *K. candel*, *A. marina*, *A. corniculatum*, and *B. gymnorrhiza* had higher abundance in environmental information processing, metabolism, and cellular processes. The results indicate that there may be specific bacteria that affect the growth and development of the mangrove plants by a species-specific means.

### Analysis of key soil properties

Rhizosphere and non-rhizosphere were analyzed. Organic matter, pH, EC, available and total nitrogen, phosphorus, and potassium among the samples appeared to be largely similar (Table 3).

**Table 3:** pH, electrical conductivity (EC), organic matter, available and total nitrogen, phosphorus, and potassium in rhizosphere and non-rhizosphere sampled from Zhangjiangkou National Mangrove Nature Reserve.

Sample	Rhizosphere of plant species	pH	EC (mS/cm)	Organic matter (g/kg)	Water-soluble nitrogen (mg/kg)	Available phosphorus (μmol/g)	Available potassium (mg/kg)	Total nitrogen (g/kg)	Total phosphorus (mg/kg)	Total potassium (g/kg)
ZQ.D	*Kandelia candel*	5.53	53.30	18.51	65.00	6.85	72.38	0.99	501.23	12.90
ZT.D	*Aegicera corniculatum*	5.59	61.40	19.19	66.90	7.51	67.85	0.95	437.83	11.44
ZB.D	*Avicennia marina*	5.43	56.00	18.81	58.80	6.53	69.96	0.96	408.37	12.46
ZL.D	*Acanthus ilicifolius*	5.53	67.90	16.90	62.80	7.06	64.59	0.77	572.34	11.80
ZM.D	*Bruguiera gymnorrhiza*	5.65	60.10	16.97	57.10	6.97	64.66	0.62	613.58	11.15
ZCK.D	Non rhizosphere	5.71	72.00	17.93	58.70	7.00	68.62	0.74	519.91	11.79

### Mental test statistical analysis at the genus level

**Figure 9:**
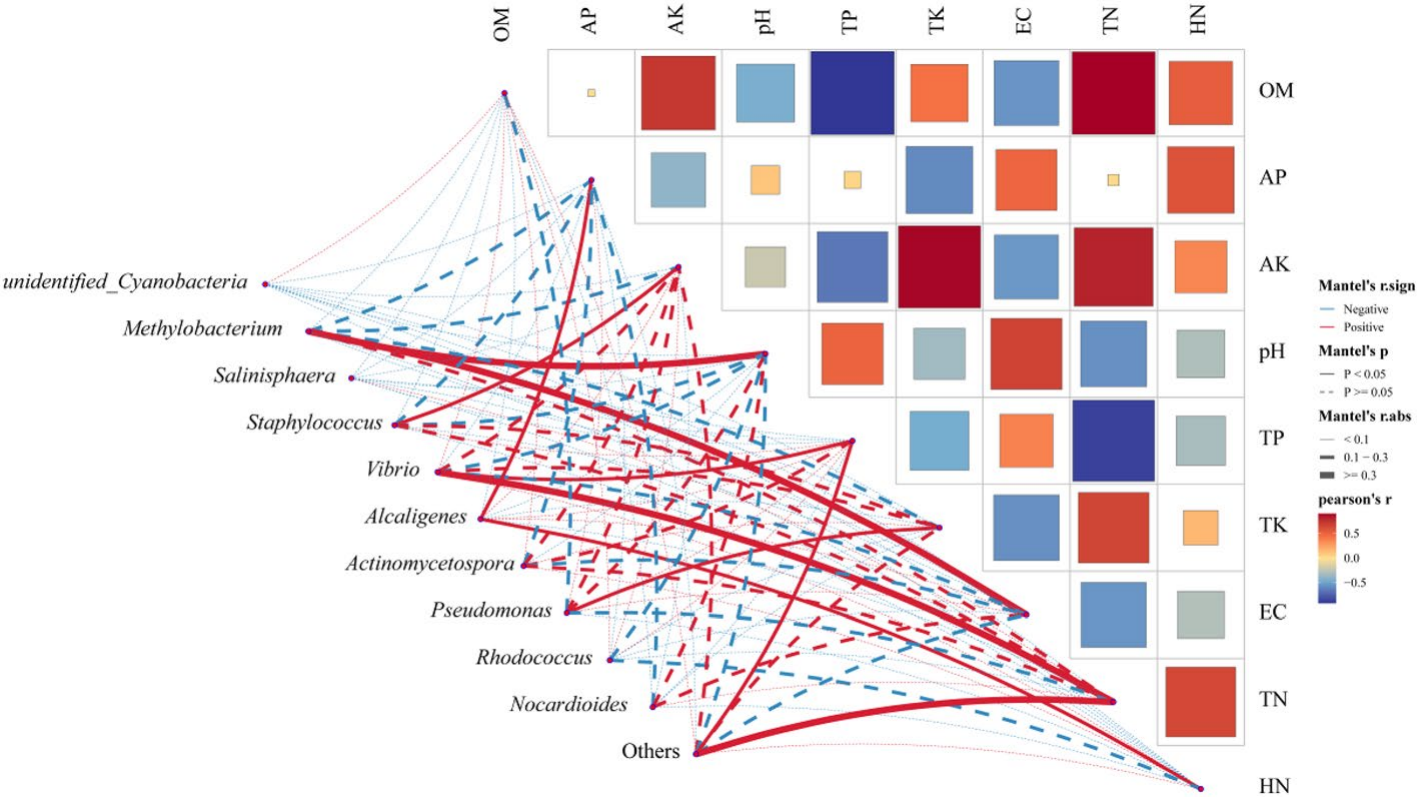
Correlation between soil main mineral elements and endophytic and rhizosphere bacteria at genus level (OM: organic matter, AP: available phosphorus, AK: available potassium, TP: total phosphorus, TK: total potassium, EC: electric conductivity, TN: total nitrogen, HN: hydrolyzed nitrogen).

To further analyze the correlation between the main mineral elements of soil and the correlation with plant rhizosphere bacteria. We performed Mantel test analysis. As shown in Fig. 9, organic matter has a very significant positive correlation with available potassium and total nitrogen, and a very significant negative correlation with total phosphorus. Available phosphorus and available potassium showed extremely significant negative correlations with the corresponding total potassium and total phosphorus respectively. In this study, the top ten bacterial genera with relative abundance were selected. The analysis results showed that the bacterial genera with significant correlation with soil chemical properties were *Methylobacterium, Staphylococcus, Vibrio, Alcaligenes* and *Pseudomonas*. The analysis showed that *Methylobacterium* showed a significant positive correlation with changes in pH and conductivity. There was a significant positive correlation between the changes in the abundance of *Staphylococcus* and the changes in available potassium content. The relative abundance of this bacterial genus was relatively high in the rhizosphere soil samples of *Kandelia candel* and *Bruguiera gymnor-rhiza*. *Vibrio* has the highest relative abundance (8.26%) in the rhizosphere soil samples of *Bruguiera gymnor-rhiza*. There was a strong correlation between the abundance changes of Alcaligenes and the changes in available phosphorus and hydrolyzable nitrogen contents. The relative abundance of this bacterial genus was the highest in the rhizosphere soil samples of *Acanthus ilicifolius*. There is a significant correlation between Pseudomonas and the total potassium content, and it is mostly distributed in the rhizosphere soil samples of *Avicennia marina*.

## Discussion

The Illumina high-throughput sequencing technology was used in this study to examine the community structure of rhizosphere bacteria and endophytic bacteria associated with five mangrove species. Endophytic bacteria were highly diverse, and the bacterial community compositions had a degree of correlation with mangrove species that was indicated by the distribution of the number of OTUs among the samples (Fig. 2). These results suggest that mangrove forests are rich in microbial resources, contributing to the productivity of mangrove ecosystems.

In general, the endophytic bacterial community of mangrove plants was dominated by Alphaproteobacteria and Gammaproteobacteria. This was like the Kandelia candel habitat in the Jiulong River Estuary in my country, which also has the highest abundance of Proteobacteria. The coexisting endophytic bacteria of the halophyte Aster tripolium L. growing in the salty meadows of central Poland are dominated by Bacillus species of the Firmicutes^16,17^. The bacteria in Alphaproteobacteria have oligotrophic properties that enable them to live in low-nutrient environments. Although mangrove ecosystems are rich in organic matter, they are deficient in nutrients overall^18^. The enrichment of Alphaproteobacteria, which includes most phototrophic bacteria, such as Rhizobacteria and Rhodospirillum, helps increase the productivity of mangrove ecosystems. Consistent with the results of multiple previous studies^4,19^, the present study found that the bacterial community in the rhizosphere of mangroves was dominated by Gammaproteobacteria and Deltaproteobacteria. Deltaproteobacteria contains most of the known sulfate-reducing bacteria, which play a key role in the deposition and cycling of nitrogen, carbon, and sulfur^20^. The bacteria in Deltaproteobacteria are anaerobic, so it could be speculated that the anaerobic conditions in mangrove soils might prompt the selection of specific microbial groups^21^.

Studies have shown that mangrove ecosystems have abundant functional bacteria resources that are related to the decomposition of organic matter and energy sources, and those bacteria have a degree of association with mangrove species. Pseudomonadales was the dominant order of endophytic bacteria in *B. gymnorrhiza* plants in the present study. Pseudomonadales contains nitrogen-fixing bacteria^22^ that can decompose glucose and are also involved in nitrate reduction^23^. Rhodospirillales and Salinisphaerales were the dominant orders of endophytic bacteria in *A. marina* plants. The bacteria in the Rhodospirillales order have anaerobic properties, can utilize light to perform anoxygenic photosynthesis for growth^19^ and may be able to increase the productivity of mangrove ecosystems. The bacteria in Salinisphaerales are considered to be halophilic and tolerant to high levels of salinity. It is speculated that the bacteria in Salinisphaerales can help improve the adaptability of mangroves to salt-rich environments^24^. Enterobacterales and Pseudonocardiales were found to be the dominant orders of endophytic bacteria in *A. corniculatum* trees. Most of the bacteria in Enterobacterales are involved in nitrate-reducing processes and can catalyze the oxidation of nitrite. Pseudonocardiales is a kind of Actinomycetes that may be related to disease prevention in mangrove plants. Bacillales, which can promote plant growth, was the dominant order of endophytic bacteria in *K. candel* plants^23^.

An important finding of this study is that there were small differences in microbial compositions between non-rhizosphere and rhizosphere and there were no specific associations between plants species and root microbes (Fig. 3). The analysis of PCoA (Fig. 5) and UPGMA cluster tree (Fig. 6) also showed that the composition of bacterial communities in the soil samples related to five mangrove species was relatively similar. This finding to some extent disapproved our hypothesis that microbial community compositions in soils should be associated with plant species. It has been well documented that in terrestrial soils plant species affect rhizosphere microbial communities^4,6,25–28^. Conversely, based on the plant-soil feedback theory, soil microbes can significantly affect plant communities by altering soil physicochemical and trophic properties or regulating plant coexistence^29–32^. Our finding may suggest that mangrove plant species differ from those grown in terrestrial soils and have a limited impact on soil microbial communities. This is consistent with the research results of Ping et al., who believe that mangrove bacteria are more susceptible to abiotic factors such as temperature, rather than cross-domain biotic factors^33^. This could be attributed to several factors: (1) soil properties in the mangrove ecosystem could be significantly affected by water properties, and the dynamics of water movement may alter soil physical and chemical properties and substantially reduce plant rhizosphere effects. Similar results were also reported by Gong et al.^32^ in which environmental conditions and historical events played an important role in shaping the bacterial communities in mangrove swamps along the coast of Beibu Gulf in Guangxi, China. (2) Five species of mangrove plants in the present study grew in similar localities in the Reserve, soil physicochemical properties and nutrient status may be similar (Table 3). A long-time adaptation to similar soil conditions may diminish the effects of plant species to soil microbes. However, there was a difference in the composition of the endophytic bacterial communities among the five species of mangrove plants, indicating that the mangrove species had a direct effect on the bacterial community structure. Thus, it is tempting to speculate that soil properties in the area where samples were collected in mangrove could play more important role than plant species in influencing bacterial compositions in the rhizosphere. Further studies in this and other mangrove nature reserve are warranted to testify this proposition.

## Conclusions

This study compared the dominant bacteria related to five species of mangrove plants and their rhizospheres in Zhangjiangkou National Mangrove Nature Reserve and analyzed the functions of the dominant bacteria. Our study showed that composition of bacterial communities in soil samples of the five mangrove species was relatively similar, which could be an indication that environmental conditions where mangrove plant growth may have substantial effects on soil microbial communities. On the other hand, there were significant differences in endophytic bacterial compositions among plant species, suggesting that plant communities can directly or indirectly influence microbial communities by altering the quantity and quality of litter^34,35^. These results provide some new information on microbial communities in the Zhangjiangkou National Mangrove Nature Reserve. As the Reserve has more than 2000 hectares, more research on microbial community compositions either as endophytes or in their rhizospheres are needed to confirm these findings.

## Materials and methods

### Sample collection

**Figure 10:**
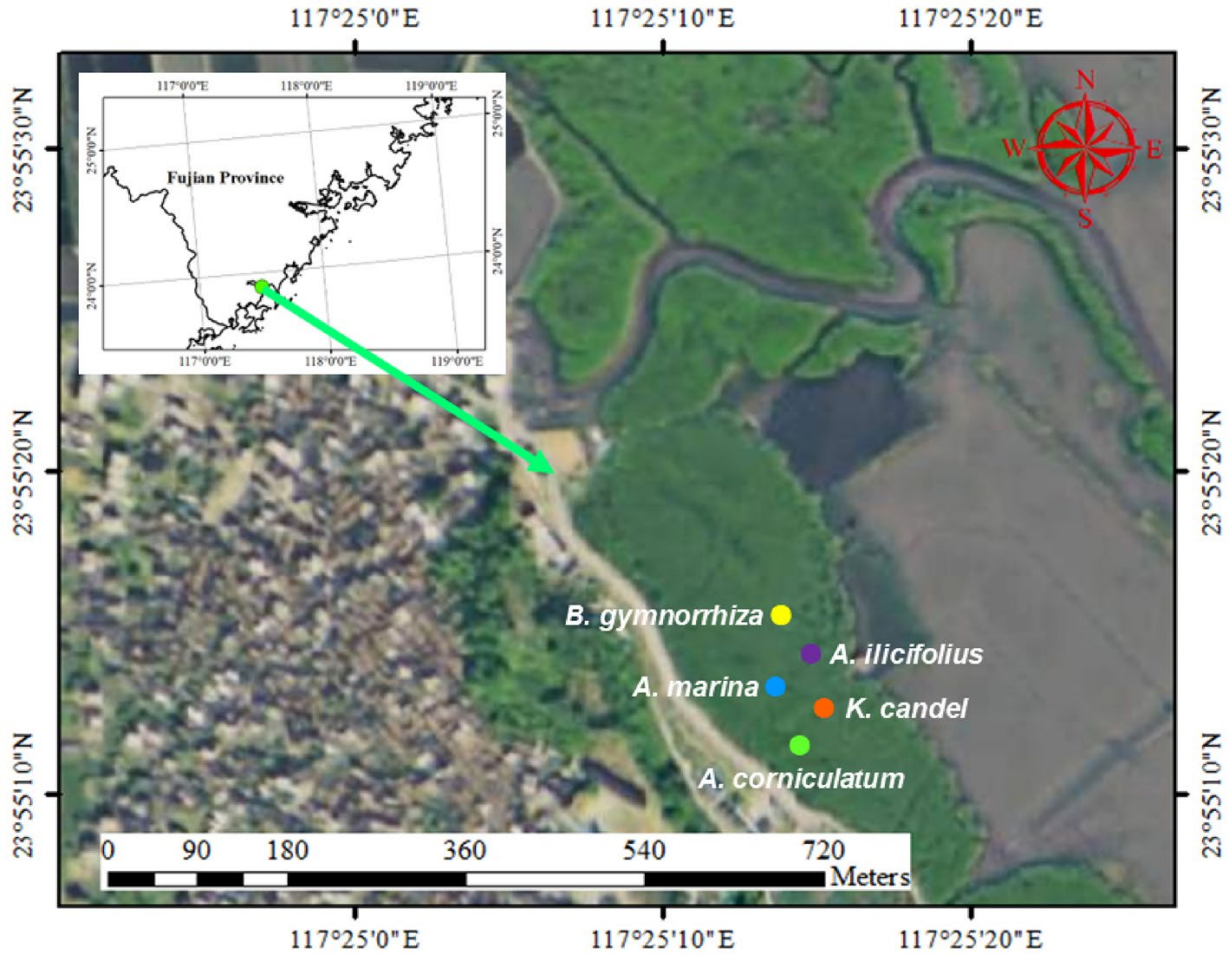
The sites where plant, rhizosphere, and soil samples were collected at Zhangjiangkou National Mangrove Nature Reserve. Roots, branches, and leaves of five mangrove species (*Kandelia candel*, *Aegicera corniculatum*, *Avicennia marina*, *Acanthus ilicifolius*, and *Bruguiera gymnorrhiza*) as well as their rhizospheres were collected (the satellite imagery was used map software: Gugu Map Downloader (official version V1.05); URL: http://www.gggis.com). Three plants were randomly selected per species. The abbreviations for *K. candel*, *A. corniculatum*, *A. marina*, *A. ilicifolius*, and *B. gymnorrhiza* were ZQ, ZT, ZB, ZL, and ZM. Root samples were labeled as ZQ.A, ZT.A, ZB.A, ZL.A, and ZM.A. Samples from branches were labeled as ZQ.B, ZT.B, ZB.B, ZL.B, and ZM.B. Leaf samples were stamped as ZQ.C, ZT.C, ZB.C, ZL.C, and ZM.C. Their rhizosphere samples were marked as ZQ.D, ZT.D, ZB.D, ZL.D, and ZM.D. The soil collected from the same area with without any roots as the bulk soil, which was used as control and labeled as ZCK.D.

The sampling took place in aforementioned Zhangjiangkou National Mangrove Nature Reserve (Fig. 10), which features a subtropical marine monsoon climate. The annual average temperature is 21.2 °C and annual precipitation is 1714 mm. In November 2020, the roots, branches, and leaves of five mangrove species (*Kandelia candel*, *Aegicera corniculatum*, *Avicennia marina*, *Acanthus ilicifolius*, and *Bruguiera gymnorrhiza*) as well as their rhizospheres were collected. The abbreviations for *K. candel*, *A. corniculatum*, *A. marina*, *A. ilicifolius*, and *B. gymnorrhiza* were ZQ, ZT, ZB, ZL, and ZM. Root samples were labeled as ZQ.A, ZT.A, ZB.A, ZL.A, and ZM.A. Samples from branches were labeled as ZQ.B, ZT.B, ZB.B, ZL.B, and ZM.B. Leaf samples were stamped as ZQ.C, ZT.C, ZB.C, ZL.C, and ZM.C. Their rhizospheres samples were marked as ZQ.D, ZT.D, ZB.D, ZL.D, and ZM.D. The soil collected from the same area with without any roots as the bulk soil, which was used as control and labeled as ZCK.D.

For sample collection, healthy and robust trees were randomly selected at the sampling sites, and their branches and leaves were collected. The roots in the soil layer depth of 20–40 cm and within 1 m distance from the trunk of each tree were collected. The soil particles less than 1 cm in size on the roots were collected as rhizosphere samples after removing impurities, such as plant residues, roots, and small stones. Three individual trees per species were randomly selected for sampling, Thus, there were 60 root, branch, leaf, and soil samples with additional three non-rhizosphere samples collected away from any trees. The collected samples were packed and sealed in sterilized bags immediately and stored at − 20 °C within 24 h after collection.

The collected soil samples, both rhizosphere and non-rhizosphere ones, were analyzed according to the methods described by Sun et al.^36^ for organic matter contents, pH and EC levels, and concentrations of available and total nitrogen, phosphorus, and potassium.

### Extraction of genomic DNA and high-throughput sequencing of amplicons of 16S rRNA gene

Genomic DNAs from root, branch, root, and soil samples were extracted using the Plant Genomic DNA Extraction Kit produced by Tiangen Biotech (Beijing, China) according to the manufacturer’s instructions. The concentration and purity of extracted genomic DNA were detected using agarose gel electrophoresis, and the qualified DNA samples were stored at − 20 °C for later use. Polymerase chain reaction (PCR) primers were designed based on the conserved regions of the bacterial 16S rRNA gene, and sequencing adapters were added to the ends of the primers. PCR amplification was performed, and the PCR products were purified, quantified, and homogenized to form a sequencing library. The constructed libraries were subjected to quality control. Qualified libraries were used to construct small fragment libraries for sequencing based on the Illumina NovaSeq sequencing platform and paired-end sequencing method. The sequencing was performed by Novogene (Beijing, China).

### Data analysis

The raw reads obtained through sequencing were processed to obtain valid sequences that were then subjected to operational taxonomic unit (OTU) clustering and the analysis of species at a 97% similarity level^37^. The rarefaction curve and hierarchical clustering of the samples were plotted using R software. The number of observed species, Shannon index, Ace index, library coverage, and phylogenetic diversity index were computed using Qiime software^38^. R software was used in the plotting of principal coordinates analysis (PCoA) results. PICRUSt analysis was used to predict the metabolic functions of the bacterial populations based on the OTU tree in the Greengene database as well as the gene information included in the OTU tree^39^.

### Determination and analysis of soil physical and chemical indicators

Determination of soil pH using potentiometric method. Soil organic matter was measured using potassium dichromate oxidation-external heating method^40^. Total N was determined using the Kjeldahl method. Hydrolyzable N was determined using the alkaline hydrolysis-diffusion method. Total P was determined using the alkali fusion-molybdenum antimony colorimetric method. The available P was measured using hydrochloric acid-sulfuric acid solution extraction method^40^. Total K was determined using alkali fusion-flame photometry. The available K was determined using ammonium acetate extraction-flame photometry. The soil conductivity was measured using the electrode method^40^. Mental test is analyzed in the “vegan” package of R (version 4.1.2)^41^.

### Statement

We have obtained the permission to collect the smples of *Kandelia candel*, *Aegicera corniculatum, Avicennia marina, Acanthus ilicifolius,* and *Bruguiera gymnorrhiza* in Zhangjiangkou National Mangrove Nature Reserve. We confirmed that all the methods were carried out in accordance with relevant Institutional guidelines and regulations.

## Data Availability

The original sequences obtained by sequencing have been uploaded to the NCBI SRA database. The relevant accession numbers was PRJNA870714.

## References

[1] Duke NC, Meynecke JO, Dittmann S, Ellison AM, Anger K, Berger U (2007). A world without mangroves?. Science.

[2] Alongi DM (2014). Carbon cycling and storage in mangrove forests. Ann. Rev. Mar. Sci..

[3] Holguin G, Vazquez P, Bashan Y (2001). The role of sediment microorganisms in the productivity, conservation, and rehabilitation of mangrove ecosystems: An overview. Biol. Fertil. Soils.

[4] Wu P, Xiong X, Xu Z, Lu C, Cheng H, Lyu X (2016). Bacterial communities in the rhizospheres of three mangrove tree species from Beilun Estuary, China. PLoS ONE.

[5] Bai S, Li J, He Z, Van Nostrand JD, Tian Y, Lin G (2013). GeoChip-based analysis of the functional gene diversity and metabolic potential of soil microbial communities of mangroves. Appl. Microbiol. Biotechnol..

[6] Gomes NCM, Cleary DFR, Pires AC, Almeida A, Cunha A, Mendoncahagler LC (2014). Assessing variation in bacterial composition between the rhizospheres of two mangrove tree species. Estuar. Coast. Shelf Sci..

[7] Chen H, Lu W, Yan G, Yang S, Lin G (2014). Typhoons exert significant but differential impacts on net ecosystem carbon exchange of subtropical mangrove forests in China. Biogeosciences.

[8] Cao M, Cui L, Sun H, Zhang X, Zheng X, Jiang J (2021). Effects of *Spartina alterniflora* invasion on soil microbial community structure and ecological functions. Microorganisms.

[9] Torsvik V, Øvreås L (2002). Microbial diversity and function in soil: From genes to ecosystems. Curr. Opin. Microbiol..

[10] Alzubaidy H, Essack M, Malas TB, Bokhari A, Motwalli O, Kamanu FK (2016). Rhizosphere microbiome metagenomics of gray mangroves (*Avicennia** marina*) in the Red Sea. Gene.

[11] Salis RK, Bruder A, Piggott JJ, Summerfield TC, Matthaei CD (2017). High-throughput amplicon sequencing and stream benthic bacteria: Identifying the best taxonomic level for multiple-stressor research. Sci. Rep..

[12] Alongi DM (1988). Bacterial productivity and microbial biomass in tropical mangrove sediments. Microb. Ecol..

[13] Fu G, Han J, Yu T, Huangshen L, Zhao L (2019). The structure of denitrifying microbial communities in constructed mangrove wetlands in response to fluctuating salinities. J. Environ. Manag..

[14] Liu M, Huiqin H, Bao S, Tong Y (2019). Microbial community structure of soils in Bamenwan mangrove wetland. Sci. Rep..

[15] Degnan PH, Ochman H (2012). Illumina-based analysis of microbial community diversity. ISME J..

[16] Hong Y, Liao D, Hu A, Wang H, Chen J, Khan S, Su J, Li H (2015). Diversity of endophytic and rhizoplane bacterial communities associated with exotic *Spartina alterniflora* and native mangrove using Illumina amplicon sequencing. Can. J. Microbiol.

[17] Szymańska S, Płociniczak T, Piotrowska-Seget Z, Złoch M, Ruppel S, Hrynkiewicz K (2016). Metabolic potential and community structure of endophytic and rhizosphere bacteria associated with the roots of the halophyte *Aster **tripolium* L. Microbiol. Res.

[18] Vazquez P, Holguin G, Puente M, Lopez-Cortes A, Bashan Y (2000). Phosphate-solubilizing microorganisms associated with the rhizosphere of mangroves in a semiarid coastal lagoon. Biol. Fertil. Soils.

[19] Zhang Y, Yang Q, Ling J, Van Nostrand JD, Shi Z, Zhou J, Dong J (2017). Diversity and structure of diazotrophic communities in mangrove rhizosphere, revealed by high-throughput sequencing. Front. Microbiol..

[20] Romero IC, Jacobson-Meyers ME, Fuhrman JA, Capone DG (2015). Phylogenetic diversity of diazotrophs along an experimental nutrient gradient in mangrove sediments. J. Mar. Sci. Eng..

[21] Thatoi H, Behera BC, Mishra RR, Dutta SK (2013). Biodiversity and biotechnological potential of microorganisms from mangrove ecosystems: A review. Ann. Microbiol..

[22] Jing H, Xia X, Liu H, Zhou Z, Wu C, Nagarajan S (2014). Anthropogenic impact on diazotrophic diversity in the mangrove rhizosphere revealed by nifH pyrosequencing. Front. Microbiol..

[23] Ge XY, Sun ZG, Li T (2016). Soil *Pseudomonas* spp., *Bacillus* spp., and microbial communities under tomato continuous cropping in greenhouse production. J. Agro-Enviro. Sci..

[24] Antunes A, Eder W, Fareleira P, Santos H, Huber R (2003). *Salinisphaera*
*shabanensis* gen. nov., sp. nov., a novel, moderately halophilic bacterium from the brine-seawater interface of the Shaban Deep, Red Sea. Extremophiles.

[25] Hooper D, Bignell D, Brown V, Brussard L, Coleman D, Giller K (2000). Interactions between aboveground and belowground biodiversity in terrestrial ecosystems: Patterns, mechanisms, and feedbacks. BioScience.

[26] Dickie IA (2007). Host preference, niches and fungal diversity. New Phytol..

[27] Wang J, Yin W, Nianpeng H, Ziqi Y, Chen C, Runguo Z (2020). Plant functional traits regulate soil bacterial diversity across temperate deserts. Sci. Total Environ..

[28] Fu Q, Shao Y, Wang S, Liu F, Tian G, Chen Y, Yuan Z, Ye Y (2022). Soil microbial distribution depends on different types of landscape vegetation in temperate urban forest ecosystems. Front. Ecol. Evol..

[29] Bever JD, Mangan S, Alexander H (2015). Maintenance of plant species diversity by pathogens. Ann. Rev. Ecol. Evol. Syst..

[30] Bachelot B, Uriarte M, Zimmerman JK, Thompson J, Leff JW, Asiaii A, Koshner J, McGuire K (2016). Long-lasting effects of land use history on soil fungal communities in second-growth tropical rain forests. Ecol. Appl..

[31] Bennett JA, Cahill JF (2016). Fungal effects on plant-plant interactions contribute to grassland plant abundances: Evidence from the field. J. Ecol..

[32] Gao GF, Li PF, Zhong JX, Shen ZJ, Chen J, Li YT, Isabwe A, Zhu XY, Ding QS, Zhang S (2019). Spartina alterniflora invasion alters soil bacterial communities and enhances soil N_2_O emissions by stimulating soil denitrification inmangrove wetland. Sci. Total. Environ..

[33] Sun P, Wang Y, Huang X, Huang B, Wang L (2022). Water masses and their associated temperature and cross-domain biotic factors co-shape upwelling microbial communities. Water Res..

[34] Chu PK, Wang S, Zeng DH (2006). Effects of single Chinese fir and mixed leaf litters on soil chemical, microbial properties and soil enzyme activities. Plant Soil.

[35] Chapman SK, Newman GS (2009). Biodiversity at the plant–soil interface: Microbial abundance and community structure respond to litter mixing. Oecologia.

[36] Sun Q, An S, Yang L, Wang Z (2004). Chemical properties of the upper tailings beneath biotic crusts. Ecol. Eng..

[37] Brannock PM, Halanych KM (2015). Meiofaunal community analysis by high-throughput sequencing: Comparison of extraction, quality filtering, and clustering methods. Mar. Genomics.

[38] Zeng XC, Guoji E, Wang J, Wang N, Chen X, Mu Y, Li H, Yang Y, Liu Y, Wang Y (2016). Functions and unique diversity of genes and microorganisms involved in arsenite oxidation from the tailings of a realgar mine. Appl. Environ. Microbiol..

[39] Ma Y, Qu ZL, Liu B, Tan JJ, Asiegbu FO, Sun H (2020). Bacterial community structure of *Pinus*
*thunbergii* baturally infected by the nematode *Bursaphelenchus*
*xylophilus*. Microorganisms.

[40] Noreen S, Yaseen T, Iqbal J, Abbasi B, Elsadek M, Eldin S, Ijaz S, Ali I (2023). Morphological and molecular characterizations of arbuscular mycorrhizal fungi and their influence on soil physicochemical properties and plant nutrition. ACS Omega.

[41] Song Y, Zhang S, Lu J, Duan R, Chen H, Ma Y, Si T, Luo M (2023). Reed restoration decreased nutrients in wetlands with dredged sediments: Microbial community assembly and function in rhizosphere. J. Environ. Manag..

